# Progress in Gliotoxin Research

**DOI:** 10.3390/molecules30183665

**Published:** 2025-09-09

**Authors:** Longqi Li, Yuxiu Liu, Qingmin Wang, Hongjian Song

**Affiliations:** State Key Laboratory of Elemento-Organic Chemistry, Frontiers Science Center for New Organic Matter, College of Chemistry, Nankai University, Tianjin 300071, Chinaliuyuxiu@nankai.edu.cn (Y.L.)

**Keywords:** gliotoxin, biosynthesis mechanism, synthesis, biological activities

## Abstract

Gliotoxin, an important fungal secondary metabolite, belongs to the class of epidithiodiketopiperazines (ETPs) and exhibits various biological activities, including immunosuppression, induction of apoptosis, and antimicrobial, antiviral, and antitumor effects. Since the initial discovery of gliotoxin and its derivatives from various fungal species, significant progress has been made in the development of isolation methods for these compounds. Understanding biosynthetic pathways and studying the functions of associated gene clusters have provided valuable mechanistic insights. To overcome the challenges of large-scale production, organic chemists have developed innovative strategies, including the construction of disulfide-containing diketopiperazine scaffolds, the synthesis of key intermediates, and the performance of enantioselective total synthesis. Recent research has further broadened our knowledge of their biological activities and molecular mechanisms, especially regarding apoptosis induction, immunomodulatory effects, antimicrobial and antitumor efficacy, structure–activity relationships, and pharmaceutical potential. This review systematically covers the evolution of gliotoxin research, from isolation techniques and biosynthetic gene cluster analysis to synthetic route development and pharmacological studies, emphasizing its diverse applications in biomedical and pesticide fields.

## 1. Introduction

Gliotoxin (GT) is an important hydrophobic fungal metabolite belonging to the class of epidithiodiketopiperazines (ETPs) [1]. ETPs are a group of bioactive secondary metabolites primarily synthesized by fungi, characterized by a common structural feature: a diketopiperazine (DKP) core structure with a disulfide bridge. GT can be produced by various fungi, including *Trichoderma viride*, *Aspergillus fumigatus*, and the deep-sea fungus *Dichotomyces cejpii* [2,3,4].

GT was first discovered in 1932 as a metabolite of the fungus *Gliocladium fimbriatum*. In 1958, Woodward proposed the structure of GT, but its specific configuration was not determined. In 1966, its precise structure was analyzed using X-ray crystallography [5], and it was named GT. In 1976, Fukuyama achieved the first total synthesis of GT using a novel solvent-dependent Michael reaction [6]. In 2012, the total synthesis method for GT had been further optimized, and Nicolaou accomplished the enantioselective total synthesis of GT and related compounds [7]. With advancements in high-resolution mass spectrometry and nuclear magnetic resonance (NMR) techniques, Dolan achieved a more precise characterization of GT’s structural features in 2015 [8].

GT exhibits significant cytotoxicity and antifungal activity [8,9,10], along with various biological functions, such as immunosuppression [11]. For instance, GT suppresses the activation, differentiation, and effector functions of immune cells by downregulating NF-κB expression, demonstrating its potential application value in organ transplantation [12]. GT also shows promising antitumor prospects by inducing apoptosis through multiple pathways. For example, it activates the Bak protein of the Bcl-2 family, triggering ROS production and activating caspase-3 [13]. It can also induce apoptosis in various tumor cells, such as neural cells and chronic lymphocytic leukemia cells [14]. Notably, GT induces apoptosis in activated hepatic stellate cells to alleviate liver fibrosis and readily penetrates the blood–brain barrier, highlighting its potential as a lead compound for anti-liver cancer drugs [15]. GT can form crosslinks with proteins and generate harmful reactive oxygen species, thereby affecting immune cell function [16]. In terms of antibacterial and antiviral activity, GT can inhibit bacterial growth by depleting metal ions [17]. Furthermore, GT exhibits significant inhibitory effects against plant pathogens, reducing the incidence and severity of plant diseases [18]. It is widely used to control rice sheath blight and other major fungal diseases, demonstrating substantial potential as an effective biopesticide [19,20]. In summary, GT has become a significant research subject in the pharmaceutical and agrochemical fields due to its diverse biological activities and broad application prospects.

In this review, we focus on the isolation methods, synthetic pathways, biological activities, and potential structure–activity relationships of GT. First, for research on isolation methods, biosynthesis, and biological activities, searches were primarily conducted in the ScienceDirect and PubMed databases. Subsequently, for studies concerning synthetic routes and structure–activity relationships, searches were primarily conducted in the SciFinder and ACS databases. Finally, the search was structured as follows: ((gliotoxin [Mesh]) AND (synthesis)) AND (activity).

## 2. Isolation

GT, an important hydrophobic fungal metabolite with a molecular mass of 326 Da, is classified as an ETP. Its biosynthesis commences with the nonribosomal peptide synthetase (NRPS) GliP, which catalyzes the condensation of phenylalanine and serine to form a dipeptidyl precursor. Subsequent intramolecular cyclization yields the characteristic diketopiperazine (DKP) core scaffold containing a disulfide bridge [21,22]. ETPs are a class of bioactive secondary metabolites primarily produced by fungi. They exhibit a broad spectrum of biological activities, including anti-proliferative, cytotoxic, immunosuppressive, antiviral, and antibacterial effects.

### 2.1. Gliotoxin Isolation

GT, the first discovered and one of the simplest ETP compounds, has been studied extensively. To date, over 100 ETP compounds, including chaetocin, aranotomin, and sporidesmin, have been isolated from natural sources [23,24,25].

*Aspergillus fumigatus*, a saprophytic fungus belonging to the genus *Aspergillus* within the family Moniliaceae, is widely distributed in soil and feedstuffs. It is the primary causative agent of animal aspergillosis [26]. Invasive aspergillosis (IA), a fungal infection resulting from the invasion of deep tissues by *Aspergillus* species [27], is predominantly caused by *A. fumigatus*. This pathogen produces a variety of immunosuppressive mycotoxins, including GT, fumagillin, fumigacin (helvolic acid), and fumigatoxin [28].

Initially identified by Weindling in 1932 from *Trichoderma virens* [29], subsequent research revealed its widespread production as a secondary metabolite in *Aspergillus* spp. (including *A. fumigatus*, *A. niger*, and *A. terreus*), *Eurotium chevalieri*, and certain *Penicillium* and *Acremonium* fungi [30,31,32,33,34,35].

In 1965, Wilkinson demonstrated GT production by *Aspergillus chevalieri* (syn. *Eurotium chevalieri*) cultured on a medium containing 6% malt extract, 4% lactose, 0–5% NaNO_3_, and trace metals [36]. The peak GT concentration was attained after seven days of surface culture at 23 °C. The active compound was extracted from the culture filtrate using chloroform. The chloroform extract was then purified by chromatography on acetic acid-deactivated alumina, yielding colorless GT crystals after crystallization from methanol or ethanol.

A 2005 study by Anitha investigated GT production by *Trichoderma virens* cultivated on natural substrates [37]. Among sugarcane bagasse, soybean meal, and rice bran, sugarcane bagasse significantly enhanced the growth and GT production of all the isolates. The strict dependence of GT production on specific cultivation conditions presents a major challenge for large-scale production. This study highlights the potential of natural matrices, particularly low-cost and readily available agricultural waste such as sugarcane bagasse, for cost-effective GT bioprocessing.

In 2019, He reported the isolation and purification of five DKPs, including GT and bisdethiobis(methylthio)gliotoxin (BmGT), from the crude extract of *Alternaria alternata* HK-25 using high-speed counter-current chromatography [38]. A total of 151.6 mg of crude extract dissolved in 5.7 mL of the solvent mixture was separated using the upper and lower phases as the mobile phase. This yielded 12.6 mg GT and 8.5 mg BmGT, respectively. HPLC analysis confirmed that the purities of both compounds exceeded 94%.

### 2.2. Gliotoxin Derivatives Isolation

**Figure 1:**
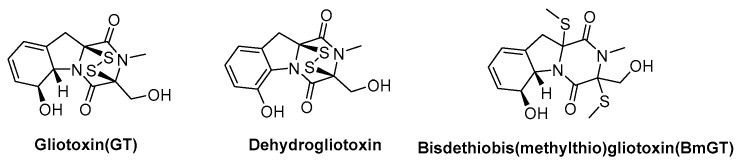
Structure of gliotoxin and gliotoxin derivatives.

GT has a diverse array of derivatives, including oxidized (dehydrogliotoxin), methylated, dethiolated (BmGT), carboxylic acid ester, and glycosylated derivatives (Figure 1). Among these, dehydrogliotoxin and BmGT are the most encountered. Dehydrogliotoxin is an oxidized GT derivative. It is frequently employed in research settings to elucidate the biosynthetic pathway of GT and investigate its mechanism of action. BmGT is an inactivated derivative of GT that is characterized by its enhanced chemical stability. BmGT is a specific diagnostic marker for detecting IA caused by *Aspergillus fumigatus*.

**Figure 2:**
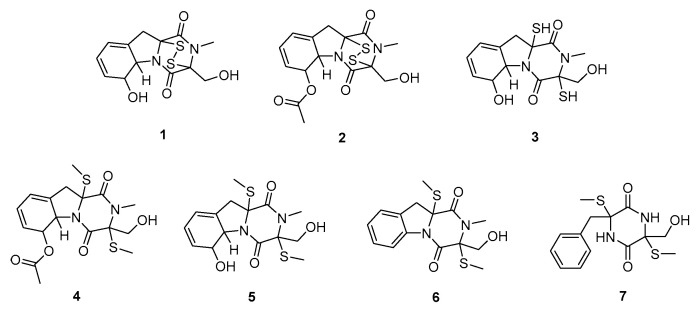
Secondary metabolites of *Neosartorya pseudofischeri*.

In 1966, Lowe reported the isolation of dehydrogliotoxin from the metabolites of *Penicillium terlikowskii* strain HLX 136 cultured on malt extract agar slants [39]. In 1986, Okamoto isolated BmGT from the metabolites of *Penicillium terlikowskii* strain 5348 [40]. A 2014 study by Liang investigated the influence of culture medium composition on the metabolite profile of the marine-derived fungus *Neosartorya pseudofischeri* (now typically classified as *Aspergillus*) [41]. Two distinct media were employed: glycerol–peptone–yeast extract (GlyPY), which contained glycerol (10 g), peptone (5 g), yeast extract (2 g), CaCO_3_ (1 g), and seawater (1 L), and glucose–peptone–yeast extract (GluPY), which contained glucose (10 g), peptone (5 g), yeast extract (2 g), and seawater (1 L), adjusted to pH 7.5. Following cultivation, the culture broths were filtered through a cheesecloth. The filtrate was extracted thrice using ethyl acetate. The combined organic extracts were concentrated under reduced pressure using low-temperature rotary evaporators. The crude extracts were subjected to chromatographic separation using silica gel columns. Target compounds were identified using techniques such as reversed-phase high-performance liquid chromatography (RP-HPLC). Metabolite production was found to be highly medium-dependent. Cultivation on GlyPY medium yielded metabolites predominantly identified as 6-acetylbisdethiobis(methylthio)gliotoxin (**4**), BmGT (**5**), and dihydrobisdethiobis(methylthio)gliotoxin (**6**). Cultivation on GluPY medium resulted in the production of GT (**1**) as the major metabolite, along with five derivatives: acetylated gliotoxin (**2**), reduced gliotoxin (**3**), 6-acetylbisdethiobis(methylthio)gliotoxin (**4**), BmGT (**5**), and bis-N-demethylgliotoxin (**7**) (Figure 2).

## 3. Synthesis

### 3.1. Biosynthesis

The structure of GT is fundamentally that of a cyclic dipeptide (DKP). However, the identification of the genes responsible for its biosynthesis was not achieved until 2005, following the completion of the *Aspergillus fumigatus* genome sequencing project [42,43]. Genes involved in the synthesis of secondary metabolites are typically located adjacent to each other within the genome and are organized into contiguous biosynthetic gene clusters (BGCs). The GT biosynthetic cluster (*gli* cluster) is located on chromosome 6. Its expression and metabolic pathway are regulated by multiple distinct proteins, indicating that GT biosynthesis is likely a multi-layered and multi-factorial process [44]. Comparative genomic analysis of *A. fumigatus* and other gliotoxin-producing fungi identified the *gli* cluster in the wild-type *A. fumigatus* AF293 strain. This cluster spans approximately 28 kb and comprises 13 genes: positive transcription factor (GliZ), 1-Aminocyclopropane-1-carboxylate (ACC) synthase (GliI), dipeptidase (GliJ), nonribosomal peptide synthetase (GliP), cytochrome P450 monooxygenase (GliC and GliF), *O*-Methyltransferase (GliM), glutathione *S*-transferase (GliG), γ-Glutamyltransferase (GliK), efflux transporter (GliA), *N*-Methyltransferase (GliN), thioredoxin reductase (GliT), and conserved hypothetical protein (GliH) [16].

**Figure 3:**
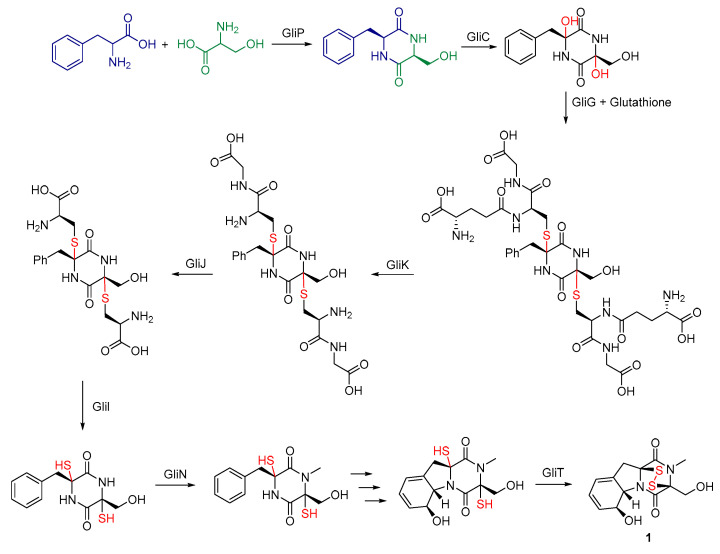
The proposed biosynthetic pathway of gliotoxin.

The mechanism of GT biosynthesis mediated by the *gli* cluster in *A. fumigatus* has been characterized [42,43,44,45]. The core steps are as follows: NRPS encoded by *gliP* catalyzes the formation of the linear dipeptide *L*-Phe-*L*-Ser. Intramolecular cyclization then generates the DKP core scaffold, cyclo(*L*-Phe-*L*-Ser) [46,47]. The dipeptide scaffold is hydroxylated by P450 monooxygenase GliF [8,48]. The hydroxyl group introduced by GliF is substituted with glutathione (GSH) by glutathione *S*-transferase GliG [49]. Subsequently, γ-glutamyltransferase GliK removes the γ-glutamyl moiety from the glutathione adduct [50]. The carboxypeptidase/dipeptidase GliJ removes the carboxyl group from the amino acid side chain [51]. This step is followed by transamination, potentially mediated by the ACC synthase-like enzyme GliI [52]. *N*-methyltransferase GliN mediates the formation of the *N*-methyl group present in mature GT [53]. Thioredoxin reductase GliT plays a crucial role in the final steps, likely involved in disulfide bond formation/rearrangement and self-protection by reducing toxic intracellular GT [54]. The *O*-methyltransferase GliM is responsible for adding *O*-methyl groups, leading to the formation of GT derivatives such as acetylated forms [8] (Figure 3).

Beyond the core enzymatic machinery, the C_2_H_2_ zinc-finger transcription factor *GliZ* serves as a master regulator of the *gli* cluster. Silencing *gliZ* abolishes GT production and significantly attenuates the pathogenicity of *Aspergillus fumigatus* in IA models [55]. Electrophoretic mobility shift assays (EMSAs) demonstrated that DcGliZ, the orthologous transcription factor in *Dichotomomyces cejpii*, binds in vitro to the promoter regions of *gliP* (pG), *gliM* (pM), and *gliN* (pN), indicating conserved regulatory mechanisms across GT-producing fungi [56]. A distinct C_2_H_2_ zinc-finger transcription factor, *GipA*, negatively regulates GT biosynthesis in *Aspergillus nidulans*. Deletion of *gipA* results in the complete abrogation of GT production in A. fumigatus [57]. GipA specifically binds to the promoter of the efflux transporter gene, *gliA*. This binding downregulates *gliA* expression, consequently reducing extracellular GT levels [58]. The C_2_H_2_ zinc-finger transcription factor MtfA acts upstream of *GliZ*. MtfA positively modulates GT biosynthesis by enhancing *gliZ* expression, thereby elevating the overall GT yield [59]. In 2023, Zhang demonstrated that the binding sequences of *GliZ* in the promoters of *gliG* were determined by DNase footprinting [60]. First, we illustrated the transcriptional regulatory mechanism of DcGliZ for gliotoxin biosynthesis in *D. cejpii* and significantly improved the yields of gliotoxins in *D. cejpii* using biosynthetic approaches.

### 3.2. Chemical Synthesis

**Figure 4:**
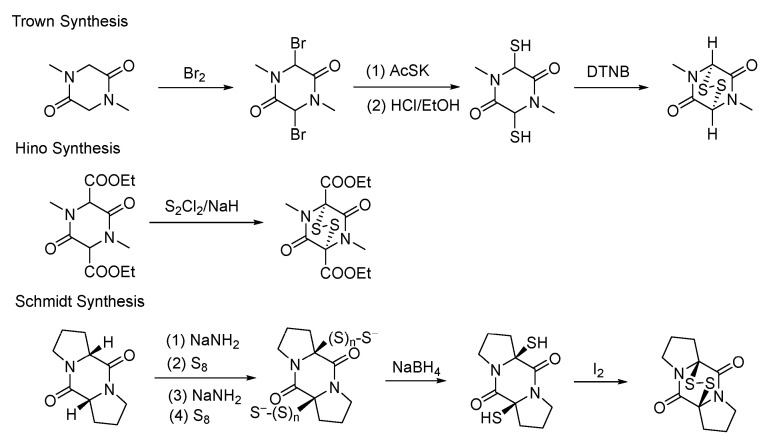
Construction of racemic epidithiodiketopiperazine system.

The unique structural features of natural products, such as GT, have attracted widespread attention from researchers in organic synthesis. One of the core challenges in synthesizing these natural products is the construction of a racemic ETP scaffold. By 1972, only three racemic ETPs had been successfully synthesized. The most historically significant synthesis was accomplished by Trown in 1968 [61]. Hino introduced a disulfide bond into the compound via the carbon anion of a 1,3-dicarbonyl system [62]. Schmidt, employing a strategy similar to Hino’s method, successfully synthesized racemic dithio-proline anhydride [63] (Figure 4).

**Figure 5:**
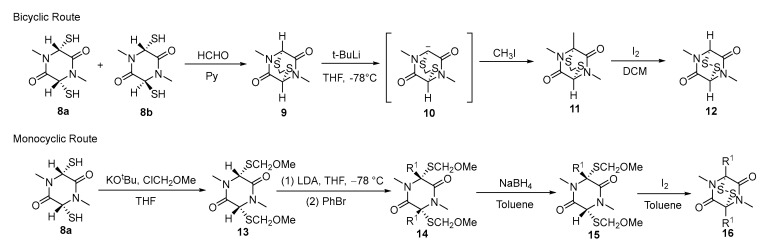
Synthesis of key intermediates of gliotoxin via bicyclic/monocyclic routes.

Gliotoxin and its derivatives, dehydrogliotoxin and hyalodendrin, all possess the ETP structure and exhibit significant antiviral, antifungal, and antibacterial activities. In 1981, Fukuyama reported the total synthesis of these three natural products: GT, dehydrogliotoxin, and hyalodendrin [64]. The study primarily designed bicyclic and monocyclic routes and synthetic approaches for constructing the key intermediate, ETP scaffold. In the bicyclic route, starting with dithiodiketopiperazines **8a** and **8b**, the reaction with formaldehyde in pyridine yielded thioacetal **9**. Subsequent treatment with *tert*-butyllithium in THF at −78 °C generated bridgehead carbanion **10**, which was then alkylated with CH_3_I to afford monomethyl thioacetal **11**. Finally, oxidation of thioacetal **11** with I_2_ yielded ETP **12**. In the monocyclic route, starting with dithiodiketopiperazine **8a**, reaction with KOtBu and ClCH_2_OMe in THF yielded dimethoxymethyl thiodiketopiperazine **13**. Compound **13** was cooled to −78 °C in THF, treated with LDA to generate the bridgehead carbanion, and then reacted with an alkylating agent to yield compound **14**. Reduction with NaBH_4_ yielded monomethyl thiodiketopiperazine **15**. Oxidation of **15** with I_2_ in toluene produced the ETP **16** (Figure 5)

The thioacetal intermediate in the bicyclic route demonstrated enhanced stability, making it more suitable for the multistep synthesis of complex compounds. The monocyclic route offers a straightforward operation and higher yield, rendering it more appropriate for the large-scale synthesis of structurally simple ETPs.

**Figure 6:**
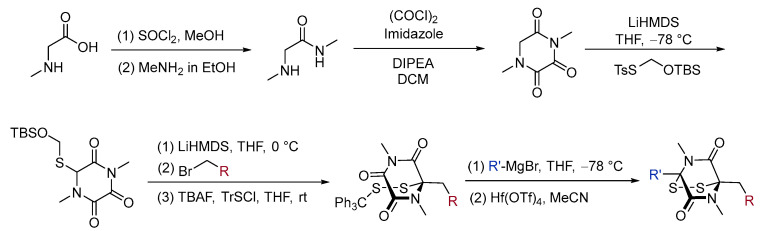
A modular construction of epidithiodiketopiperazines.

In 2019, Snaddon discovered an operationally straightforward and modular synthesis method for ETPs [65]. Starting from sarcosine, diversification via a modular three-step alkylation/disulfide formation/Grignard addition-ring closure sequence provided a range of ETP analogs in useful yields on a preparative scale (Figure 6).

**Figure 7:**
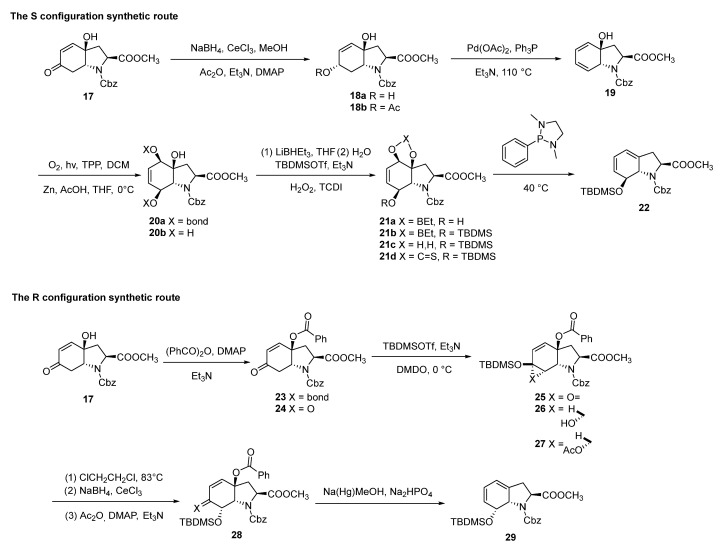
Synthetic routes for the two stereoisomers of 7-hydroxy-2,3,7,7a-tetrahydroindole ring.

In 1996, Sundberg reported two stereodivergent routes for the synthesis of the 7-hydroxy-2,3,7,7a-tetrahydro-1*H*-indole ring of gliotoxin from tyrosine via oxidative cyclization, affording the 7-(*R*) and 7-(*S*) enantiomers, respectively [66]. Starting from tyrosine, oxidative cyclization afforded compound **17**. Compound **17** was reduced at the C6 carbonyl using NaBH_4_-CeCl_3_ to yield **18a**. Compound **18a** underwent acetylation and a Pd(OAc)_2_-mediated elimination reaction to yield diene **19**. Compound **20a** (presumably a typo in the original, likely referring to **19**) was subjected to photooxygenation to form endoperoxide **20a**, which was subsequently reduced with zinc in acetic acid to afford triol **20b**. Compound **20b** was converted to boronate ester **21a**, and the C7 hydroxy group was protected with TBDMSOTf to yield **21b**. Compound **21b** underwent hydrolysis of the boronate ester with H_2_O_2_ to afford compound **21c**. Compound **21c** was converted to thiocarbonate **21d**, which was then reacted with 1,3-dimethyl-2-phenyl-1,3,2-diazaphospholidine to furnish target compound **22** in a 47% yield. Similarly, starting from tyrosine, compound **17** was converted to benzoate **23**, which was transformed into silyl enol ether **24**. Compound **24** was oxidized with DMDO to yield epoxysilane **25**, which underwent thermal rearrangement upon heating in 1,2-dichloroethane to afford ketone **26**. Compound **26** was subjected to reduction with NaBH_4_-CeCl_3_, acetylation, and reduction with sodium amalgam to yield compound **29** (58% yield). Compounds **22** and **29** were characterized by ^1^H and ^13^C NMR, confirming that their structures were consistent with the expected ones, definitively establishing the 7-(*S*) and 7-(*R*) stereochemistry, respectively (Figure 7).

**Figure 8:**
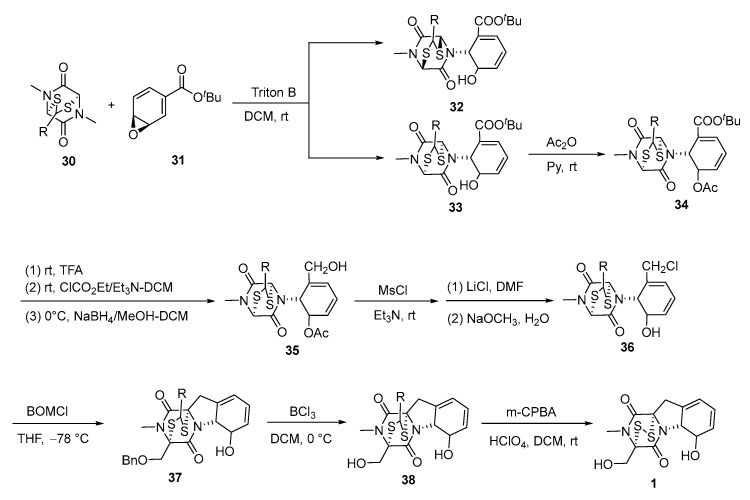
Solvent-dependent Michael reaction in the total synthesis of gliotoxin.

In 1976, the first total synthesis of gliotoxin, featuring a solvent-dependent Michael reaction as a key step, was reported by the same group. Starting from glycine sarcosine anhydride, thioacetal **30** was synthesized in six steps with an overall yield of 30% [6]. 4-tert-Butoxycarbonylbenzaldehyde (**31**) reacted with **30** in DCM containing benzyltrimethylammonium hydroxide at room temperature for a short period. The major product was alcohol **32** (45% yield), and the minor product was epimer **33** (15% yield). The ratio of alcohols **32** and **33**, resulting from the Michael reaction, was dependent on the solvent and reaction time. Alcohol **33** was converted to acetate **34** with a 90% yield. This was then transformed into hydroxymethyl derivative **35** in three steps with an overall yield of 70%. Subsequently, **35** was subjected to mesylation (MsCl/Et_3_N/rt), treated with lithium chloride in DMF, and then hydrolyzed with NaOCH_3_ in MeOH-DCM at room temperature, affording chloride **36** in a 95% overall yield. Phenyl lithium was added slowly to a mixture of **36** and an excess of chloromethyl benzyl ether in THF at −78 °C, and the reaction was monitored by thin-layer chromatography. This yielded benzoyl benzimidazole acetal **37** with an isolated yield of 45% yield. Subsequently, **37** was reacted with boron trichloride in DCM at 0 °C to afford benzimidazole acetal **38** in 50% yield. The oxidation of **38** with m-chloroperbenzoic acid (m-CPBA), followed by treatment with perchloric acid in DCM at room temperature, furnished racemic gliotoxin **1** in 65% yield (Figure 8).

**Figure 9:**
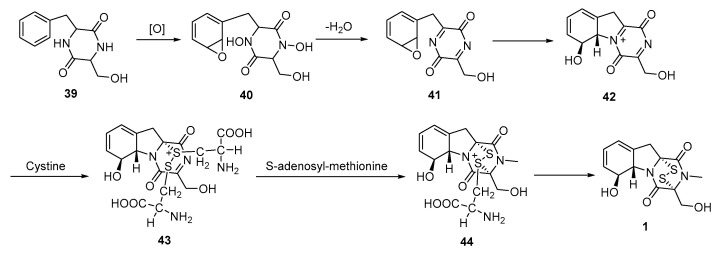
Synthesis of gliotoxin via stereochemically controlled disulfide addition reaction.

In 1980, Herscheid reported a novel approach to gliotoxin (**1**) that involved the stereochemically controlled addition of a disulfide to a di-*N*-hydroxy species [67]. Phenylalanine-serine anhydride **39** was oxidized to di-*N*-hydroxy species **40**, which underwent dehydration to generate imide **41**. Subsequently, the phenoxide species presumably attacked the N atom of the phenylalanine residue, and concurrently with or following this ring-opening of the anhydride, cysteine reacted with the iminium ion to introduce sulfur, resulting in compound **43**. A β-elimination reaction and *N*-methylation occur within the cysteine fragment, generating a second iminium ion. This ion presumably reacts with disulfide species to form **44**. Finally, a second β-elimination reaction occurred, and gliotoxin (**1**) was detected in a portion of the product (Figure 9).

**Figure 10:**
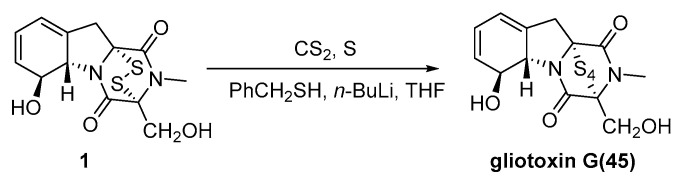
Synthesis of gliotoxin G from gliotoxin as a starting material.

In 1986, gliotoxin G (GT-G) was first identified as a minor metabolite of *A. fumigatus* that exhibits immunosuppressive activity, although its production via the natural pathway is low. In the same year, Gordon devised a method for synthesizing GT-G from GT [68]. GT was first dissolved in carbon disulfide (CS_2_). Excess sulfur (960 mg) and a small amount of Li-thiobenzolate (0.03 mmol) were added. The reaction proceeded at room temperature, and its progress was monitored by ^1^H NMR spectroscopy. After completion, the mixture was poured into a silica gel column. Excess sulfur was eluted with DCM, followed by diethyl ether to obtain GT-G, GT, and trisulfide derivatives. GT-G was isolated with a 60% yield (Figure 10).

**Figure 11:**
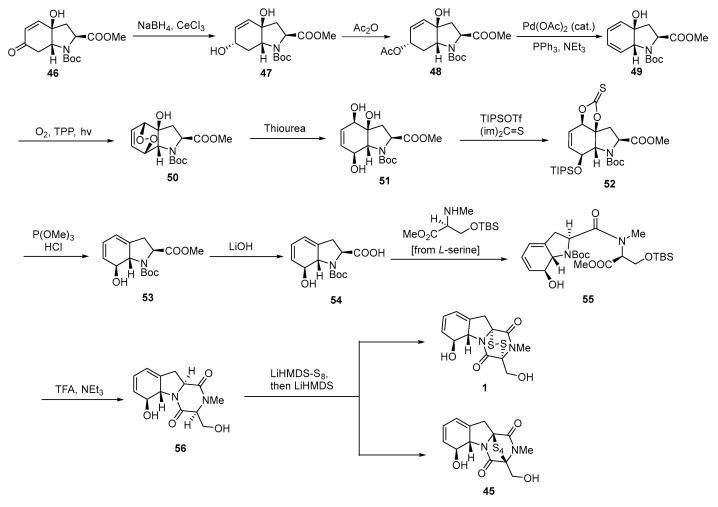
Enantioselective total synthesis of gliotoxin and gliotoxin G.

In 2012, Nicolaou investigated synthetic methods for ETPs, epitetrathiodioxopiperazines, and bis(methylthio)diketopiperazines and achieved the enantioselective total synthesis of GT, GT-G, and related compounds [7]. Starting from the tyrosine-derived hydroxy enone *N*-Boc methyl ester **46**, **46** was reduced to diol **47** via Luche reduction using NaBH_4_ and CeCl_3_, with a 99% yield. Acetylation of **47** afforded hydroxy acetate **48** in 91% yield. A palladium-catalyzed elimination reaction of **48** using Pd(OAc)_2_ and PPh_3_ converted it to hydroxy diene **49** in 86% yield. Photooxygenation of **49** using O_2_ and TPP as the photosensitizer yielded hydroxy endoperoxide **50** in 73% yield. Reduction of **50** with thiourea yielded triol **51** in an 84% yield. The monosilylation of **51** with TIPSOTf afforded intermediate **52** in 96% yield. Deoxygenation of **52** with P(OMe)_3_ yielded intermediate **53** in an 82% yield. Desilylation of intermediate **53** with HCl furnished the final product **53** in a 98% yield. Further reaction: Hydrolysis of the methyl ester group in **53** to carboxylic acid **54** was achieved with LiOH in 99% yield. The amidation coupling of **54** with an *L*-serine derivative using HATU, HOAt, and DIPEA afforded amide **55** in an 88% yield. Removal of the Boc protecting group in **55** with TFA, followed by cyclization using Et_3_N, yielded tricyclic diketopiperazine **56** in a 63% yield. Finally, a sulfurization reaction using LiHMDS-S_8_ and LiHMDS directly converted **56** to **1** (GT) and **45** (GT-G), with yields of 23% and 33%, respectively (Figure 11).

Approximately one-third of the global population is affected by tuberculosis, which causes nearly two million deaths annually. The emergence of drug-resistant strains has intensified the demand for economically viable treatment options for these infections. Dehydrogliotoxin, a compound isolated from *P. terlikowskii*, inhibits macrophage phagocytosis at concentrations like those of GT and is considered a potential candidate for anti-tuberculosis therapy.

**Figure 12:**
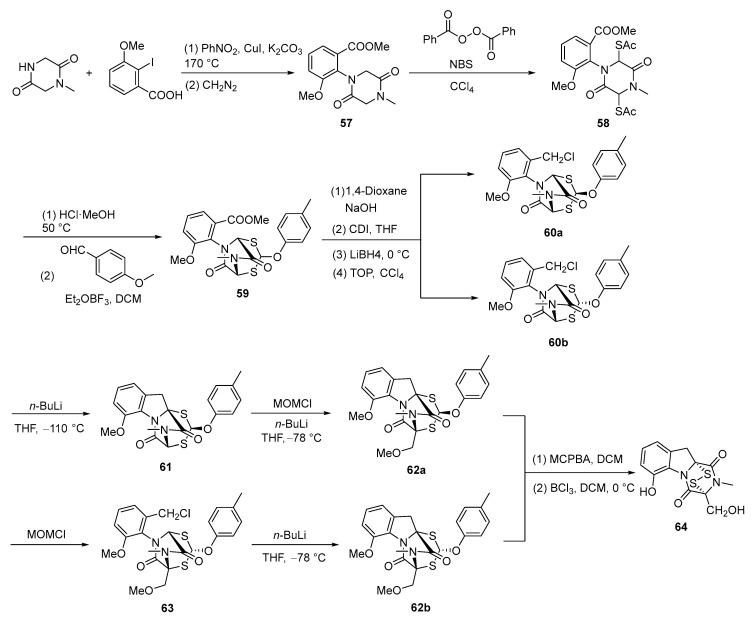
Total synthesis route of dehydrogliotoxin.

In 1973, Kishi reported the first total synthesis of dehydrogliotoxin [69]. Starting from 1-methylpiperazine-2,5-dione and 2-iodo-3-methoxybenzoic acid, esterification with diazomethane was performed in nitrobenzene in the presence of copper(I) iodide and potassium carbonate. Heating at 170 °C for 40 min afforded **57** in 50% yield. The oxidation of **57** with NBS and benzoyl peroxide (BPO) in carbon tetrachloride, followed by a reaction with potassium thioacetate in methylene chloride, yielded **58**. Treatment of **58** with methanolic hydrogen chloride at 50 °C to remove the glyoxylic residue, followed by reaction with anisaldehyde in methylene chloride containing a trace of boron trifluoride etherate at room temperature, afforded anisaldehyde adduct **59**. Subjecting compound **59** to a four-step sequence yielded a mixture of compounds **60a** and **60b** in 41% yield. Separation of **60a**, followed by treatment with *n*-butyllithium at −110 °C and subsequent quenching with acetic acid, yielded compound **61** in 38% yield. Alkylation of **61** with chloromethyl methyl ether (MOMCl) at −78 °C afforded compound **62a** in 61% yield. Alternatively, separation of **60b** followed by alkylation with MOMCl yielded compound **63**. Subsequent treatment of **63** with *n*-butyllithium at −78 °C afforded compound **62b** in an 80% yield. Treatment of compounds **62a** and **62b** with mCPBA in methylene chloride, followed by reaction with boron trichloride (BCl_3_) in methylene chloride at 0 °C, yielded dehydrogliotoxin (**64**). The structure of **64** was confirmed by NMR, IR, UV, and mass spectrometry. The yield in the final step was 20% (Figure 12).

**Figure 13:**
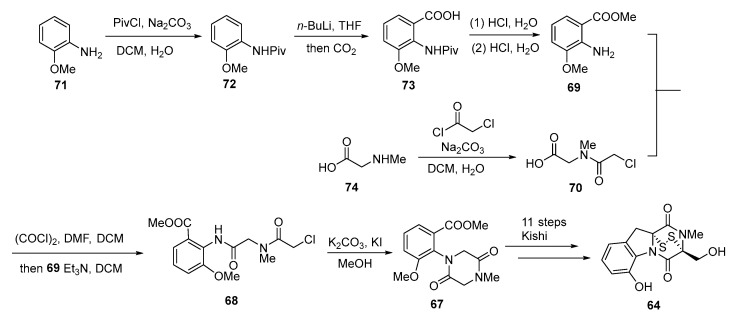
Synthesis route for the key intermediates of gliotoxin derivatives.

In previous research, the key intermediate for the synthesis of dehydrogliotoxin by Kishi was DKP **67**, which is the product of an Ullmann-type coupling between iodinated aryl **65** and DKP **66**. In 2011, McMahon optimized the existing synthetic route starting from this key intermediate and designed a high-yielding, economical, and scalable total synthesis route for dehydrogliotoxin [70]. Retrosynthetic analysis revealed that aniline **69** and acid **70** were important precursors for synthesizing key intermediate **67**. Using *o*-anisidine (**71**) and carnitine (**74**) as starting materials, aniline **69** and acid **70** were synthesized. Aniline **69** and acid **70** were then reacted with (COCl)_2_ in DMF and DCM to generate diamide **68**. Finally, the intramolecular cyclization of diamide **68** produced key intermediate **67**, and **64** was subsequently obtained via Kishi’s synthetic method (Figure 13).

## 4. Bioactivity

Fungal secondary metabolites represent critical genetic traits for survival and adaptation within specific ecological niches. Genes encoding these metabolites are typically organized into biosynthetic gene clusters (BGCs) [71], which are frequently located within subtelomeric regions near the chromosome termini [72,73]. These metabolites serve diverse functions, including signaling, antagonism, inflicting damage on plant and animal hosts, promoting infection, protecting fungi from host immune cells, and facilitating the acquisition of essential nutrients [74,75,76,77,78,79,80].

GT is one of the most extensively studied fungal secondary metabolites. This sulfur-containing mycotoxin, which belongs to the ETP class, is produced by diverse fungi. A defining structural feature is the disulfide bridge that spans the diketopiperazine ring. This bridge enables crosslinking with proteins via cysteine residues and participates in redox cycling between the reduced and oxidized states. During cycling, GT generates deleterious reactive oxygen species (ROS). This is widely accepted as a key mechanism underlying GT toxicity [16].

The diverse effects of GT on various cell types and microorganisms primarily stem from its immunosuppressive and immunomodulatory activities [81]. In vitro studies have demonstrated that GT has the capacity to inhibit multiple processes critical to immune cell activation, differentiation, and effector functions [42]. Downregulation of NF-κB expression and induction of apoptosis [81]. Impairment of neutrophil function, including inhibition of chemotaxis [82,83] and phagocytosis by polymorphonuclear neutrophils (PMNs) and macrophages [83,84]. Inhibition of cytotoxic T cell activation [85]. Suppression of interferon-gamma (IFNγ) production by CD4^+^T lymphocytes [86]. Beyond immunosuppression, GT induces apoptosis in mammalian cells [87,88], a process accompanied by ROS generation and disruption of mitochondrial membrane integrity [89,90].

### 4.1. Apoptosis

Apoptosis, also known as programmed cell death (PCD), is a genetically controlled process that is essential for development, tissue homeostasis, and responses to damage or disease in multicellular organisms. It differs fundamentally from necrosis, an unregulated form of cell death that is typically caused by acute external injury. Extensive research has established that GT promotes apoptosis through multiple molecular pathways.

GT activates the pro-apoptotic Bcl-2 family protein Bak. This activation triggers excessive ROS generation. Release of apoptogenic factors from mitochondria. Activation of caspase-3 executes the final stages of apoptosis. The pathological accumulation of ROS is a pivotal mechanism underlying GT-induced apoptosis. Furthermore, ROS amplifies the apoptotic cascade initiated by apoptogenic factors and cytochrome [89]. Following cellular uptake and induction of apoptosis, GT is predominantly released in its oxidized form. This extracellular GT subsequently enters adjacent cells and propagates apoptotic signaling pathways. This amplification loop enables a maximal cytotoxic impact, even at low GT concentrations [91]. Experimental evidence has demonstrated that GT activates the c-Jun N-terminal kinase (JNK) signaling pathway in murine fibroblasts, murine alveolar epithelial cells, and human bronchial epithelial cells. JNK activation leads to Bak- and caspase-dependent apoptosis in the cells. This mechanism is considered a critical factor in driving disease progression in IA [13].

In 2006, Julian identified the mitochondrial protein Bak as a critical factor in GT-induced apoptosis [92]. Initial experiments monitoring mouse embryonic fibroblasts (MEFs) revealed that GT induced apoptosis. Bak, Bax, and Bid proteins all belong to the Bcl-2 family. During apoptosis, Bak and Bax undergo conformational changes, resulting in the exposure of their N-terminal domains [93,94,95]. Subsequently, experiments established that GT-induced apoptosis was dependent on Bak. Only Bak undergoes a conformational change in this process, and this effect is independent of Bid and Bax proteins, caspase activation, or ROS production. However, GT-induced mitochondrial and Forte membrane damage depends on the generation of ROS. Furthermore, GT-induced caspase-3 activation and cytochrome c release were dependent on Bak activation and ROS generation. Finally, experiments demonstrated that mice lacking the mitochondrial protein Bak exhibited resistance to IA, further confirming that Bak is a key protein mediating the virulence of *A. fumigatus* towards host cells.

The induction of apoptosis in activated hepatic stellate cells (aHSCs) by GT highlights its potential application in the treatment of liver fibrosis. In 2007, Anselmi discovered that GT induces apoptosis and necrosis in rat Kupffer cells in vitro and in vivo under conditions without oxidative stress, achieved through the inhibition of caspases and serine proteases [96]. The experiments involved injecting GT into Kupffer cells isolated from normal livers and into rats with CCl_4_-induced liver cirrhosis. Subsequent analysis demonstrated that 0.3 μM GT induced apoptosis in cultured Kupffer cells within 1 h of treatment. This apoptotic process was associated with mitochondrial cytochrome c release, caspase-3 activation, and adenosine triphosphate (ATP) depletion. Importantly, the inhibition of caspase-3 and serine proteases accelerated GT-induced necrosis. In cirrhotic rats, in vivo GT injection caused apoptosis in Kupffer cells, hepatic stellate cells (HSCs), and hepatocytes. This study indicates that GT non-specifically induces hepatocyte death in fibrotic livers. Molecular modifications of GT may be responsible for its ability to selectively target and eliminate activated stellate cells.

In 2016, Kim demonstrated that GT induces apoptosis in HT1080 human fibrosarcoma cells by increasing intracellular ROS levels and inhibiting the NF-κB signaling pathway, revealing its potential as an antitumor agent [12]. HT1080 cells were cultured in DMEM supplemented with 10% fetal bovine serum (FBS), and cell viability was assessed using the MTT assay. Flow cytometric analysis of apoptosis induced by GT extracted from *Aspergillus fumigatus* showed a significant increase in the proportion of cells in the late apoptotic stage. Nuclear and cytoplasmic proteins were extracted from the cells. Western blot analysis of proteins associated with the NF-κB signaling pathway revealed that GT inhibited the phosphorylation and degradation of IκB-α and reduced the nuclear translocation of the NF-κB p65 subunit, thereby suppressing its activation. Finally, intracellular ROS levels were measured using dihydroethidium (DHE) staining and the Muse Oxidative Stress Kit. The results indicated that ROS levels were significantly increased in the GT-treated cells.

### 4.2. Inhibits Host Immune Responses

It has now been confirmed that GT inhibits host immune responses, including the phagocytic and microbicidal functions of macrophages and polymorphonuclear cells, activation and degranulation of mast cells, and the function of antigen-presenting cells. Studies have indicated that alveolar macrophages and neutrophils are the primary phagocytic cells responsible for clearing invasive *Aspergillus fumigatus*. Alveolar macrophages can phagocytose dormant conidia of *A. fumigatus*; however, this typically does not elicit an inflammatory response. Only when these dormant conidia begin to germinate and expose β-glucans is NADPH oxidase activated, enabling the killing of the conidia. Neutrophils serve as a backup defense line and can act against hyphae. They can form aggregates around conidia to inhibit their growth or release antimicrobial substances, such as lactoferrin, to suppress hyphal growth. Additionally, neutrophils can trigger a respiratory burst, releasing ROS and granular material to eliminate the hyphae [97].

One well-established mechanism by which GT exerts its immunosuppressive effects is through the inhibition of NF-κB activation. It has been reported that GT is a potent inhibitor of the activation of the transcription factor nuclear factor kappa B (NF-κB) in T cells and B cells, which is induced by various stimuli. The p50/p65 heterodimer is the most abundant member of the Rel/NF-κB family of transcription factors. This family controls the expression of numerous genes associated with immune response, inflammation, and cell proliferation. Dysregulation of NF-κB has been linked to cellular transformation and maintenance of a high anti-apoptotic threshold in transformed cells. NF-κB activity is regulated by its sequestration in the cytoplasm via the inhibitor of kappa B alpha (IκBα), which is susceptible to proteasomal degradation. Research has demonstrated that this effect of GT is achieved by preventing the proteasomal degradation of IκBα [98].

NADPH oxidase is present in phagocytes and generates large amounts of ROS to oxidatively modify microorganisms, representing a common microbicidal mechanism that is crucial for host defense against pathogens. In 2000, Yoshida investigated the effect of the mycotoxin GT on human neutrophil NADPH oxidase activity and found that GT inhibits O_2_^−^ generation by neutrophils, thereby impairing their immune function [99]. Experiments revealed that GT undergoes redox cycling with intracellular reductants, reducing cytochrome c oxidation and consequently inhibiting NADPH oxidase activity. Concurrently, the study found that pretreatment of neutrophils under hypoxic conditions enhanced the inhibitory effect of GT on NADPH oxidase. This indicates that GT can inhibit O_2_^−^ production by neutrophils, particularly before NADPH oxidase activation by PMA. Additionally, the S-S bridge structure of GT was identified as the primary cause of its toxicity towards NADPH oxidase at low oxygen concentrations. Conversion of GT to its bis-dithiol derivative by adding the reducing agent dithiothreitol (DTT) protected the cells from the loss of oxidase activity.

In 2004, Tsunawaki studied how the *A. fumigatus* metabolite GT inhibits the assembly of human respiratory burst NADPH oxidase [100]. ROS are key weapons for polymorphonuclear leukocytes (PMN) to kill A. fumigatus, and GT significantly inhibits phorbol myristate acetate (PMA)-stimulated O_2_^−^ generation. The research found that GT prevents the phosphorylation of p47phox and concurrently blocks the cytoskeletal integration and membrane translocation of subunits, including p47phox, p67phox, and p40phox, all of which are critical for the assembly of active NADPH oxidase. The experiments demonstrated that *A. fumigatus* inhibits PMN immune function by targeting the assembly process of NADPH oxidase with GT, thereby weakening the bactericidal capacity of neutrophils and aiding in the survival and proliferation of *A. fumigatus* within the host.

In 2005, Nishida explored how the fungal metabolite GT inhibits the activation process by targeting flavocytochrome b558 within the human NADPH oxidase complex [101]. GT inhibits NADPH oxidase activation by targeting flavocytochrome b558, a core component of the NADPH oxidase complex. In the experiments, acetylated cytochrome c and iodonitrotetrazolium violet (INT) were used as electron acceptors to avoid interference from side reactions. The results indicated that GT directly impairs a critical site in the electron transfer of flavocytochrome b558 that must be engaged before NADPH oxidase activation, thereby affecting the electron transfer process rather than acting directly on the assembly process of NADPH oxidase.

In 2006, Kupfahl demonstrated, using a designed low-dose infection mouse model of IA, that deletion of the *gliP* gene resulted in impaired GT production by *A. fumigatus*, but the virulence of *A. fumigatus* itself towards the host was unaffected [47]. BALB/c mice were treated with cyclophosphamide (150 mg/kg) and cortisone acetate (200 mg/kg), followed by infection via injection of conidia from *A. fumigatus* (ATCC 46645) and a *gliP* mutant strain generated by gene knockout of the ATCC 46645 strain. Results from the low-dose infection mouse model of IA showed that when mice were infected with the *gliP* mutant, GT was not detected in the mouse lungs using HPLC and tandem mass spectrometry. However, the virulence of the *gliP* mutant strain was not different from that of the corresponding wild-type strain. This indicates that the nonribosomal peptide synthetase GliP is essential for GT production by *A. fumigatus*. Furthermore, GT is not required for the pathogenicity of *A. fumigatus* in immunocompromised mice.

In 2007, Sugui found that the *A. fumigatus* (B-5233) *gliP* mutant exhibited attenuated virulence compared to the B-5233 strain in hydrocortisone-induced immunosuppressed mice [59]. *A. fumigatus* B-5233 is a highly virulent clinical isolate that does not require growth on vitamin-supplemented media. The first step of the GT biosynthetic pathway is catalyzed by nonribosomal peptide synthetase (NRPS) encoded by the *gliP* gene; the *gliP* mutant metabolizes GT. Results from lung sections of strain-infected mice showed that mice infected with B-5233 exhibited multifocal bronchopneumonia with necrosis, neutrophil infiltration, and airways filled with necrotic tissue debris and fungal hyphae at 72 h. However, lesions in mice infected with the *gliP* mutant did not extend beyond the bronchioles. The experiment demonstrated that the *gliP* mutant had a reduced ability to damage host cells and a significantly diminished capacity to suppress the oxidative burst of neutrophils, resulting in markedly lower toxicity to mice than the wild-type strain. Furthermore, experiments comparing two mouse strains, 129/Sv and BALB/c, indicated differences in host susceptibility to *A. fumigatus* infection.

In 2008, Spikes found that GT produced metabolically by *A. fumigatus* (Af293) exerted a virulent effect only on non-neutropenic mice and on *Drosophila melanogaster* with functional phagocytes, suggesting that the primary targets of GT are neutrophils or other phagocytes [102]. First, the *gliP* mutant was obtained by gene knockout and culture of the Af293 strain. Since the NRPS GliP catalyzes the condensation of serine and phenylalanine, the first step in GT biosynthesis, the *gliP* mutant lost the ability to produce GT metabolically, while its growth rate was consistent with the wild-type. Subsequently, the *gliP* mutant was transfected with pBC-phleo-*gliP*. HPLC-MS/MS analysis confirmed detectable GT production in both Af293 and the transfected *gliP* mutant. Mouse virulence testing demonstrated that GT exhibited significant virulence in non-neutropenic mice. Additionally, *D. melanogaster* lacks neutrophils but possesses functional circulating phagocytes [103]. Tl mutants susceptible to lethal infection by Af293 were selected as a complementary biological model. Virulence testing on the Tl mutants proved that *A. fumigatus* capable of metabolically producing GT exhibited greater virulence towards *D. melanogaster*. The study indicated that neutrophils or other phagocytes are the targets of GT.

In 2019, König reported that GT specifically inhibits the epoxide hydrolase activity of LTA4H, reducing LTB_4_ formation and thereby inhibiting neutrophil aggregation and function [82]. Experiments using mouse and rat models, in which peritonitis and pleurisy were induced, respectively, assessed the effect of GT on neutrophil recruitment and leukotriene LTB_4_ formation. It was found that GT significantly inhibited induced neutrophil recruitment and LTB_4_ formation in both mouse and rat models, without affecting the levels of other lipid mediators. Using human neutrophils and monocytes to investigate the effect of GT on LTB_4_ formation, the results indicated that GT specifically inhibited the epoxide hydrolase activity of LTA4H, reducing LTB_4_ formation, but had no effect on the aminopeptidase activity of LTA4H. Furthermore, additional experiments revealed that the inhibitory effect of GT requires an intracellular reducing environment, as its dithiol form is necessary to effectively inhibit LTA4H activity. GT-treated neutrophils also exhibited nuclear fragmentation, suggesting that it may influence neutrophil function by inducing apoptosis.

### 4.3. Antineoplastic Activity

The apoptosis-inducing mechanism of GT exerts its anti-cancer effects by inducing cancer cell apoptosis, primarily through the oxidative stress pathway, mitochondrial pathway, and Bcl-2 family protein regulation mechanism, and manifests its anti-cancer functions via the following three actions. GT can significantly inhibit the proliferation of various tumor cells. For example, in esophageal cancer cell lines EC9706, EC109, and KYSE-150, different concentrations of GT (0, 1, 5, 10, 20, 40 μM) significantly inhibited the proliferation of these cells [104]. Through the apoptosis-inducing mechanism, GT can induce apoptosis in various tumor cells. For example, in neuronal cells, treatment with 300 nmol/L GT for 12 h resulted in varying degrees of apoptosis observed under electron microscopy [105]. GT may also reverse tumor cell resistance to traditional chemotherapeutic drugs by inducing non-apoptotic cell death (such as paraptosis). For example, Lercanidipine (Ler), Loperamide (Lop), Nutlin-3, and SNIPER (TACC3), when combined with bortezomib (Btz), enhanced the anti-cancer activity of bortezomib and overcame tumor cell resistance to bortezomib by inducing tumor cell paraptosis [106].

Chronic lymphocytic leukemia (CLL) is a common adult leukemia characterized by the abnormal proliferation of B cells in the bone marrow and peripheral blood. CLL cells resist apoptosis due to support from the bone marrow microenvironment, specifically bone marrow stromal cells. The Notch signaling pathway is a highly conserved mechanism of intercellular communication, widely present in multicellular organisms. It mediates signal transduction through direct cell-to-cell interactions, regulating cell fate decisions, proliferation, differentiation, apoptosis, and tissue homeostasis. It plays a significant role in the survival and proliferation of CLL cells.

In 2011, Rainer found that GT inhibits Notch activity, inducing apoptosis in CLL cells and overcoming the supportive effect of primary bone marrow stromal cells [14]. Preliminary experiments revealed that GT can rapidly induce apoptosis in CLL cells. Its mechanism of action is closely related to the inhibition of the Notch2 signaling pathway. Following treatment, CLL cells exhibited inhibited Notch2 transcriptional activity, leading to a significant downregulation in the expression of downstream anti-apoptotic genes (such as CD23/FCER2). Secondly, using a co-culture model of CLL cells and bone marrow stromal cells, it was found that GT not only induces apoptosis in CLL cells but also overcomes the protective effect of bone marrow stromal cells on CLL cells.

Tumor multidrug resistance (MDR) is one of the primary causes of treatment failure in cancer therapy. Ferroptosis is an emerging type of programmed cell death that provides multiple potential therapeutic targets for cancer treatment and is becoming a hotspot in oncology and anti-cancer research. The core mechanism of ferroptosis involves lipid peroxidation induced by Fe^2+^ or lipoxygenases (LOXs), coupled with a drastic decrease in glutathione (GSH) content. Concurrently, the inactivation of glutathione peroxidase 4 (GPX4) leads to massive accumulation of reactive oxygen species (ROS), ultimately destroying cellular structures and resulting in cell death [107,108,109].

In 2023, Chen found that GT can induce cellular ferroptosis by downregulating the expression of SUV39H1 [110]. Designed cell experiments demonstrated that GT exhibits potent antitumor activity in non-small cell lung cancer cells H1975 and breast cancer cells MCF-7, with IC_50_ values of 0.24 µM and 0.45 µM, respectively. GT also induced ferroptosis by increasing intracellular Fe^2+^ and ROS levels while simultaneously decreasing GSH and ATP levels. Malondialdehyde levels were significantly elevated in treated cells, indicating enhanced lipid peroxidation. Further research revealed that GT could downregulate the expression of SUV39H1 in esophageal cancer cells, thereby inducing cellular ferroptosis. In esophageal cancer tissues, high expression of SUV39H1 is associated with poor prognosis. Additionally, using label-free quantitative proteomic analysis, the study found that GT treatment induced significant changes in intracellular protein expression, involving reorganization of the intracellular Zn uptake system and ribosomal proteins. Finally, utilizing an esophageal cancer xenograft mouse model, the results showed that GT significantly reduced tumor weight and volume, further validating its antitumor efficacy. GT demonstrates potent antitumor activity and warrants an in-depth investigation to develop novel antitumor therapies based on cellular ferroptosis.

The Mitogen-Activated Protein Kinase (MAPK) plays a crucial role in maintaining cell wall integrity. Previous studies have demonstrated that *Aspergillus fumigatus* MpkA is involved in the regulation and production of GT [111]. In 2024, Patrícia investigated the involvement of the *A. fumigatus* MAPK MpkA in GT production and self-protection [112]. During GT biosynthesis, GliT:GFP and GtmA:GFP were primarily localized to the cytoplasm and vacuoles. Under GT self-protection conditions, these proteins were additionally observed in structures resembling an endomembrane-like network and endocytic/exocytic vesicles. Cultured MpkA mutant strains failed to produce either GT or its derivative BmGT, indicating MpkA is essential for GT biosynthesis. Further research revealed that MpkA, through regulating GliT and GtmA, interacts with several protein kinases, phosphatases, and enzymes involved in glutathione metabolism and oxidative stress responses during GT production. Additionally, the study demonstrated that other protein kinases and peroxisomes participate in GT’s self-protection mechanism.

### 4.4. Antiviral Activity

In HIV-1-infected individuals receiving combination antiretroviral therapy (cART), the virus persists in a latent form within the body, leading to rapid viral rebound upon treatment cessation. Therefore, developing therapeutic strategies capable of reactivating the latent virus and eliminating infected cells is crucial for an HIV-1 cure.

In 2020, Mateusz reported that GT, as a novel latency-reversing agent (LRA), effectively reverses HIV-1 latency and exhibits favorable safety and synergistic effects at low concentrations [113]. Using Jurkat cell line models (J-Lat A2 and 11.1) and primary CD4+ T cell models, the study demonstrated that GT directly targets La-related protein 7 (LARP7). By disrupting the 7SK snRNP complex, GT induces degradation of 7SK RNA and releases active P-TEFb, thereby activating HIV-1 transcription. Furthermore, GT showed no significant impact on the viability or proliferative capacity of CD4+ T cells and CD8+ T cells at low concentrations (20 nM) and did not induce T cell activation. Finally, GT demonstrated synergistic latency reversal effects when combined with the histone deacetylase (HDAC) inhibitor SAHA and the BAF inhibitor CAPE.

### 4.5. Antifungal and Antibacterial Activity

GT, as an important fungal secondary metabolite, demonstrates significant potential value in the field of biological control. Certain species of the genus *Trichoderma* have been utilized for controlling plant pathogens. GT production is associated with the antifungal activity of these biocontrol agents, and its effect may be enhanced synergistically with cell wall-degrading enzymes (CWDEs). Studies have shown that GT was identified as a highly active compound in *T. virens* HZA14, capable of causing colony collapse and degradation of *Phytophthora capsici*, thereby significantly reducing the incidence and severity of pepper blight [18].

Due to years of persistent overuse and misuse of antibiotics, major bacterial pathogens have developed antimicrobial resistance (AMR), leading to reduced efficacy of many existing antibiotics and consequently increased patient mortality. The fungal nonribosomal peptide compound GT is known to possess antibacterial activity. Its reduced form, dithiol gliotoxin (DTG), acts as a chelator for Zn, Cu, and Fe. In 2023, Downes found that GT likely exerts growth-inhibitory effects on other bacteria by depleting metal ions [17]. Firstly, it was observed that the growth inhibition of GT against both Gram-positive and Gram-negative bacteria could be reversed by the addition of Zn^2+^ or Cu^2+^. Simultaneously, GT enhanced vancomycin-mediated growth inhibition of *Enterococcus faecium*, and this inhibitory effect could also be alleviated by adding Zn^2+^. Further proteomic analysis revealed that the presence of GT caused significant reorganization of the intracellular proteome in *E. faecium*, particularly changes in the expression of Zn uptake systems and ribosomal proteins. Therefore, it was further hypothesized that GT inhibits bacterial growth by depleting intracellular Zn or reducing Zn bioavailability and may function further by interfering with Cu homeostasis. These findings provide a theoretical basis for developing novel antibacterial strategies.

## 5. Structure–Activity Relationships

Lysine-specific demethylase 1 (LSD1) is a highly conserved protein whose functional diversity is supported by its complex structure, enabling interactions with numerous cellular proteins. Human LSD1 protein comprises 852 amino acids and encodes three main domains: (1) The *N*-terminal SWIRM domain (Small α-Helical Domain), composed of approximately 85 residues, is crucial for protein–protein interactions and contributes to protein stability and the formation of the substrate-binding region. (2) The *C*-terminal Amino Oxidase-Like (AOL) domain exhibits approximately 20% sequence similarity to FAD-dependent oxidases. The AOL and SWIRM domains interact to form a globular conformation. (3) The centrally projecting TOWER domain is formed by the FAD-binding domain and the substrate-binding domain via the AOL domain [114]. Currently, LSD1 is recognized as a promising therapeutic target for cancer treatment [115,116,117]. Studies have also confirmed that LSD1 is closely associated with the development of gastric cancer and may be a potential target for its treatment [118]. To date, numerous LSD1 inhibitors have been discovered [119,120], including both reversible and irreversible LSD1 inhibitors. Some of these LSD1 inhibitors are undergoing clinical trials for the treatment of leukemia and solid tumors [121,122,123,124].

In previous research, the metabolite GT, isolated from the soil-derived fungus *Aspergillus fumigatus* ZSS02, was found to exhibit a certain inhibitory effect on LSD1 activity. In 2023, Shan investigated the anti-proliferative activity of 6-heterocyclic carboxylate derivatives of GT against gastric cancer cells. They designed and synthesized a series of 6-heterocyclic carboxylate derivatives of GT as novel LSD1 inhibitors and conducted biological evaluations in human gastric MGC-803 and HGC-27 cells [125]. First, molecular docking using the MOE 2015 software predicted the potential binding modes of GT and its derivatives with LSD1. It was found that GT and its derivatives may form hydrogen bonds with amino acid residues within the FAD-binding pocket of LSD1, potentially via disulfide bonds, thereby stabilizing the binding conformation. All synthesized derivatives in the designed series exhibited comparatively stronger LSD1 inhibitory activity than GT itself. Among them, compound **75e** demonstrated the most potent activity, with an IC_50_ value of 62.40 nM.

**Figure 14:**
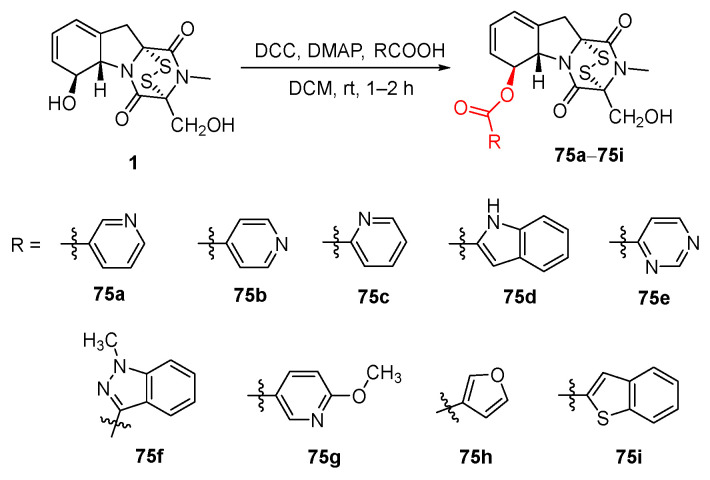
Synthesis route and the structures of **75a**–**75i**.

In 2023, Shan designed and synthesized 6-heterocyclic carboxylate derivatives of GT as novel LSD1 inhibitors [125]. Through optimization of the culture medium and fermentation conditions, it was found that under the following conditions: soluble starch 21.3 g/L, peptone 15.8 g/L, K_2_HPO_4_ 1.2 g/L, initial pH 4.5, inoculum size 2%, liquid volume 150 mL/500 mL, temperature 28 °C, agitation speed 200 r/min, *L*-serine 4.0 g/L, *L*-phenylalanine 1.6 g/L, fermentation time 96 h, and addition of a small amount of surfactant Tween-80, the yield of GT isolated from *Aspergillus fumigatus* ZSS02 was increased to 208 mg/L. Using GT as the starting material dissolved in DCM, with DCC as the dehydrating agent and DMAP as the catalyst, reaction with different heterocyclic carboxylic acids at room temperature yielded a total of nine derivatives (**75a**–**75i**) (Figure 14). This series of compounds exhibited significantly superior inhibitory activity against LSD1 compared to the parent compound GT and could be used in the preparation of antitumor drugs for clinical treatment of human esophageal, gastric, lung, colorectal, and breast cancers.

The 6-heterocyclic carboxylate derivatives introduce a heterocyclic carboxylate group (pyridine, pyrimidine, indole) at the 6-position while retaining the core disulfide bond to maintain binding affinity to LSD1. Experimental results indicated that both GT and its derivatives form hydrogen bonds with the FAD-binding pocket of LSD1 via the disulfide bond (salt bridge with Arg120), a structure critical for activity. However, the 6-position ester group is a tolerant site amenable to modification for activity optimization, allowing the introduction of diverse heterocycles. Nine derivatives (**75a**–**75i**) were synthesized via mild esterification reactions, achieving high yields. Among them, the pyrimidine carboxylate derivative showed over a hundred-fold increase in LSD1 inhibitory activity (IC_50_ = 62.40 nM), likely due to more favorable electronic or steric effects of the pyrimidyl group. Compound **75e** demonstrated potent anti-proliferative effects in gastric cancer cells (MGC-803, HGC-27; IC_50_ ≈ 0.3 μM), inhibited colony formation and migration, and induced apoptosis. Furthermore, **75e** suppressed cancer cell metastasis by regulating epithelial–mesenchymal transition (EMT) markers (upregulating E-cadherin, downregulating Snail/Vimentin).

**Figure 15:**
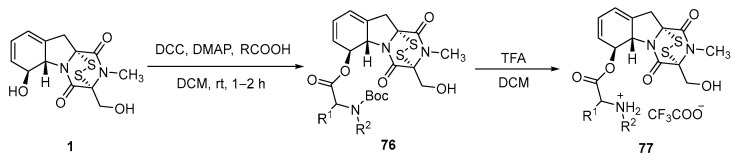
Synthesis route of *L*-amino acid-6-gliotoxin ester trifluoroacetate compound.

In 2021, Shan reported a series of *L*-amino acid-6-gliotoxin ester trifluoroacetate compounds exhibiting enhanced stability and higher antitumor activity [126]. GT was dissolved in DCM and subjected to an esterification reaction with various *N*-Boc-*L*-amino acids catalyzed by DCC and DMAP. Purification by column chromatography afforded intermediate **76**. Subsequently, the intermediate was dissolved in DCM, and an excess of TFA was added to remove the Boc protecting group and form the trifluoroacetate salt (Figure 15). The reaction mixture was concentrated, followed by precipitation with isopropyl ether to afford the final product **77**. The inhibitory activities of the resulting derivatives against LSD1 were tested. The results showed IC_50_ values significantly lower than those of GT, indicating stronger inhibitory activity.

**Figure 16:**
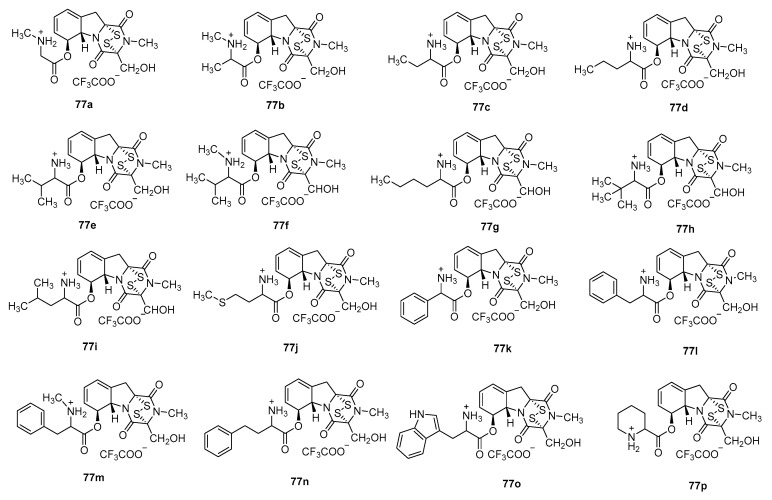
Gliotoxin derivatives *L*-amino acid-6-gliotoxin ester trifluoroacetate compound.

The *L*-amino acid-6-gliotoxin ester trifluoroacetates retained the core ETP scaffold and the crucial disulfide bond active center of GT, ensuring their binding capability to the LSD1 target. Among this class of derivatives in LSD1 activity assays, compound **77a** exhibited the strongest activity (IC_50_ = 40.86 nM). This potency enhancement is potentially attributable to the methyl side chain (*N*-methylglycine, sarcosine) increasing the compound’s hydrophobicity or due to steric effects. The 2-phenylglycine derivative **77k** showed the next highest activity (IC_50_ = 76.38 nM), possibly through enhanced target binding via π-π stacking or hydrophobic interactions. The norvaline derivative **77d** (IC_50_ = 373.7 nM) and the tryptophan derivative **77o** (IC_50_ = 124.4 nM) exhibited lower activity (Figure 16). The reasons for this may be that the long alkyl chain and the bulky indole ring could cause steric hindrance or reduce compound solubility, thereby interfering with target binding.

In IA, GT is primarily produced by *Aspergillus fumigatus* and serves as a significant virulence factor. BmGT is a methylated, detoxified derivative of GT. It is generated via catalysis by the methyltransferase GtmA. BmGT exhibits significantly reduced toxicity but superior stability compared to GT, making it a potential IA-specific biomarker. This is particularly advantageous in non-immunosuppressed patients [127]. As previously mentioned, GT’s activity depends on its disulfide group. BmGT, however, loses the ability to generate ROS due to the methylation of its thiol groups, thereby reducing its toxicity. Concurrently, methylation alters the molecule’s lipophilicity and enhances its stability, rendering BmGT more suitable as a diagnostic biomarker. In clinical diagnostics, the combined detection of BmGT and galactomannan (GM) significantly improves the specificity (93%) and positive predictive value (100%) of IA diagnosis. This approach is especially valuable for atypical hosts.

## 6. Conclusions

GT is a small-molecule compound with a molecular weight of 326 Da, belonging to the class of ETPs. In biological systems, it is synthesized from a cyclic dipeptide formed by the condensation of phenylalanine and serine, catalyzed by the nonribosomal peptide synthetase GliP. Initially discovered in 1932, its precise structure was determined in 1966 via X-ray crystallography. Early isolation methods primarily relied on extraction from fungal cultures, such as *T. viride* and *A. fumigatus*. These methods typically involve complex extraction and purification steps, resulting in low yields. With technological advancements, modern isolation methods have become more efficient and precise. When combined with genetic engineering techniques, these methods can significantly increase both the yield and purity of isolated GT.

The biosynthesis of GT originates from simple amino acid substrates and advances through a coordinated sequence of enzymatic reactions comprising cyclization, strategic functionalization via hydroxylation, glutathionylation, and methylation, followed by successive tailoring modifications. This pathway culminates in the elaboration of the molecule’s distinctive and complex disulfide bridge architecture. Notably, the biosynthetic machinery is not solely dedicated to structural assembly but is intrinsically coupled with a self-detoxification mechanism mediated by GliT, thereby demonstrating the high efficiency and adaptive strategy inherent to fungal secondary metabolism. Chemical synthesis methods for GT and its derivatives are diverse, involving various organic synthesis techniques and approaches. From early solvent-dependent Michael reaction-based total syntheses to modern enantioselective total syntheses, routes for synthesizing GT and its derivatives have been continuously optimized, achieving higher yields and stereoselectivity.

GT possesses potent biological functions. It induces apoptosis through multiple pathways, including the activation of the Bak protein within the Bcl-2 family generation of ROS, and the activation of caspase-3, ultimately leading to apoptosis. GT can downregulate the expression of NF-κB, thereby suppressing immune cell activation, differentiation, and effector functions, exerting immunosuppressive effects. GT also exhibits antibacterial activity by depleting metal ions, indicating potential value for pesticide applications. It additionally demonstrates antiviral activity, such as inhibiting HIV-1 latency by disrupting the 7SK snRNP complex and activating HIV-1 transcription. Moreover, GT induces apoptosis in tumor cells through multiple pathways, including the oxidative stress pathway, the mitochondrial pathway, and the regulation of Bcl-2 family proteins.

In conclusion, GT, as an important fungal secondary metabolite, exhibits diverse biological functions and broad application prospects, with its antitumor activity receiving particularly widespread attention in recent years. Future research should focus on the following: exploring more efficient, economical, and environmentally friendly methods for GT isolation and synthesis; utilizing microbial heterologous expression systems to produce GT and its derivatives for large-scale production; and designing novel GT derivatives through structure–activity relationship analysis and molecular docking technology to enhance their bioactivity and application value. These research directions warrant further in-depth investigation.

## Data Availability

Data have been stored in the library repository of Nankai University, Tianjin, China.

## Abbreviations

The following abbreviations are used in this manuscript:

GT	Gliotoxin
BmGT	Bisdethiobis(methylthio)gliotoxin
DKP	Diketopiperazine
ETPs	Epidithiodiketopiperazines
IA	invasive aspergillosis
